# Impact of Genetic Variability in ACE2 Expression on the Evolutionary Dynamics of SARS-CoV-2 Spike D614G Mutation

**DOI:** 10.3390/genes12010016

**Published:** 2020-12-24

**Authors:** Szu-Wei Huang, Sorin O. Miller, Chia-Hung Yen, Sheng-Fan Wang

**Affiliations:** 1 Model Development Section, Basic Research Laboratory, Center for Cancer Research, National Cancer Institute, Frederick, MD 21702, USA; szu-wei.huang@nih.gov (S.-W.H.); sorin.miller@nih.gov (S.O.M.); 2 Graduate Institute of Natural Products, College of Pharmacy, Kaohsiung Medical University, Kaohsiung 80708, Taiwan; 3 Drug Development and Value Creation Research Center, Kaohsiung Medical University, Kaohsiung 80708, Taiwan; 4 National Natural Product Libraries and High-Throughput Screening Core Facility, Kaohsiung Medical University, Kaohsiung, 80708, Taiwan; 5 Center for Tropical Medicine and Infectious Disease, Kaohsiung Medical University, Kaohsiung 80708, Taiwan; 6 Department of Medical Laboratory Science and Biotechnology, Kaohsiung Medical University, Kaohsiung 80708, Taiwan; 7 Department of Medical Research, Kaohsiung Medical University Hospital, Kaohsiung Medical University, Kaohsiung 80708, Taiwan

**Keywords:** SARS-CoV-2, spike, D 614G, evolution, genetic variation, ACE2

## Abstract

The severe acute respiratory syndrome coronavirus 2 (SARS-CoV-2) spike (S) glycoprotein D614G mutation became the predominant globally circulating variant after its emergence in the early coronavirus disease 2019 (COVID-19) pandemic. Studies showed that this mutation results in an open conformation of the S glycoprotein receptor-binding domain (RBD), and increased angiotensin 1-converting enzyme 2 (ACE2) binding and fusion, which result in an increase in SARS-CoV-2 transmissibility and infectivity. Dynamic tracking of SARS-CoV-2 showed that the D614G variant became predominant after emergence in Europe and North America, but not in China. The current absence of selective pressures from antiviral treatment suggests that the driving force for viral evolution could be variations in human population genetics. Results show that ACE2 expression is higher in Asian populations than that in European, North American, and African populations. This supports the idea that lower ACE2 expression is a driving force in the positive selection for the D614G mutation. This study suggests that the dynamics of the SARS-CoV-2 D614G mutation during the early-to-mid pandemic is associated with enhanced transmission efficiency in populations with lower ACE2 expression. Understanding the role that human genetic diversity plays in the adaptive evolution of SARS-CoV-2 may have an important impact on public health and measures to control the pandemic.

## 1. Introduction

The recently identified severe acute respiratory syndrome coronavirus 2 (SARS-CoV-2), which emerged in late 2019, is responsible for the pandemic of coronavirus disease 2019 (COVID-19) [1,2,3,4]. This pandemic is ongoing, and the global number of confirmed SARS-CoV-2 cases continues to rise. The rapid spread allows for the continuous propagation of various mutations. A recent study estimated that the mean evolution rate of SARS-CoV-2 ranges from 1.729 × 10^−3^ to 1.8266 × 10^−3^ substitutions per site per year [5]. A genomic report of SARS-CoV-2 showed 767 synonymous and 1352 nonsynonymous mutations in 4254 genome sequences, with the *ORF1ab*, *S*, and *N* genes being more frequently mutated than others are [6]. The patterns of these nonsynonymous mutations in SARS-CoV-2 were found to differ across geographic regions [7]. Positive selection contributes to the evolution of SARS-CoV-2, with genes that display high diversity, adjusting to allow for the protein to adapt to new environments [6,8]. As there is currently no vaccine selection, human genetic diversity is suggested to be a driving force in the adaptive evolution of SARS-CoV-2. The continuous exposure of the virus to new environments in infected hosts creates selective pressure. These different human genetic populations may correlate to different genetic patterns of SARS-CoV-2 as a result of selective pressure on SARS-CoV-2 transmission.

One of the predominant mutations, D614G, is located in the spike (S) glycoprotein in SARS-CoV-2 and was found by a current study to display increased virulence [9,10,11,12,13]. This mutation was not detected during the early pandemic, but became prominent outside China [9]. The most important receptor for the SARS-CoV-2 S glycoprotein was identified as angiotensin 1-converting enzyme 2 (ACE2). Genetic variations in ACE2 and its protein expression levels could provide the driving force for viral evolution, thereby causing the positive selection for D614G in the SARS-CoV-2 S glycoprotein. Investigating correlations between genetic variation in global populations with viral infectivity or with clinical outcomes could provide great insights for measuring the risk for public health, controlling the pandemic, and developing precision-medicine strategies. In this study, we compiled and analyzed the current knowledge on the impact of genetic variation in ACE2 on the susceptibility to SARS-CoV-2. Our results strongly suggest a possible correlation between the SARS-CoV-2 S glycoprotein D614G mutation and diversity within ACE2 expression levels in human genetic populations.

The SARS-CoV-2 S glycoprotein contains a furin recognition cleavage site (polybasic cleavage site, PRRAR) which provides efficient proteolytic processing into S1 and S2 [14]. The SARS-CoV-2 receptor-binding domain (RBD) located in the S1 domain binds to the host cell ACE2 receptor, while S2 functions as the membrane fusion subunit [15,16]. The D614G mutation in the S glycoprotein can affect SARS-CoV-2 infectivity by affecting the RBD structure, S1/S2 subunit interaction, viral entry, and immune response [12]. A molecular virology study by Zhang and colleagues showed that the D614G mutation in the SARS-CoV-2 S glycoprotein can decrease S1 shedding and increase S glycoprotein incorporation into the virion, thereby enhancing SARS-CoV-2 infectivity [9]. However, according to a recent study, whether more S glycoprotein is incorporated into the SARS-CoV-2 D614G virion is still controversial [13]. Another study by Becerra-Flores and colleagues found that patients infected with SARS-CoV-2 containing the D614G mutation have a higher case fatality rate [17]. This mutation was also found to be associated with higher viral load in the upper respiratory tract in patients, and with increased infectivity in multiple pseudotyped experiments [12], which was also confirmed in animal models [11,13]. Notably, the structure of the RBD in SARS-CoV-2 D614G variant displayed a more open conformation, which may increase the probability of ACE2 binding and fusion steps [10]. These studies underline the importance of continuing to monitor the impact of the D614G mutation on the COVID-19 pandemic.

## 2. Materials and Methods

### 2.1. Frequency Analysis of D614 and G614 Variants across Geographic Regions

A total of 121,895 SARS-CoV-2 sequences from 1 January to 12 October 2020 from the Los Alamos National Laboratory website (COVID-19 Viral Genome Analysis Pipeline [12], https://cov.lanl.gov/) were analyzed to determine weekly frequencies of D614 and G614 variants. On the basis of the website, the emergence date of the G614 variant was determined using two criteria. First, the cumulative sequences reached 15, and both variants were represented at least three times; second, there were at least 15 sequences available at least two weeks after the first emergence date. The numbers of SARS-CoV-2 sequences for analysis are: global, *n* = 121,895; China, *n* = 821; Europe, *n* = 70,153; North America, *n* = 30,712; South Asia, *n* = 3076; and Africa, *n* = 2193.

### 2.2. ACE2 Expresion Levels in Different Genetic Human Populations

The cumulative ACE2 expression score was analyzed in each individual by 21 ACE2 genetic polymorphisms that significantly affect its expression level according to a current study [18]. Expression quantitative trait loci (eQTL) in Genotype-Tissue Expression (GTEx, release V8; https://gtexportal.org/home/) [19] were used to determine the genetic polymorphisms that affect ACE2 expression. First, all genetic polymorphisms that affect ACE2 expression across all cell and tissue types were determined. Second, the genetic polymorphisms in linkage disequilibrium were removed. Lastly, 21 ACE2 genetic polymorphisms were identified. The normalized effect size (NES) of each genetic polymorphism was obtained from eQTL data. The ACE2 expression level of an individual was defined as the sum of the levels of all 21 ACE2 genetic polymorphisms. The ACE2 expression score was calculated using the formula: $\sum_{i=1}^{x}\left(n_{i}\times NES_{i}\right)$, where *n* is the number of alternative alleles (reference allele = 0, heterozygous = 1, homozygous alternative allele = 2), *x* is the number of evaluated polymorphisms in ACE2, and NES is the effect of the alternative allele relative to the reference allele [18]. A total of 2504 individuals were included for analysis from the 1000 Genomes Project phase 3 release [20]. Five geographic regions were used in the analysis of ACE2 expression: South Asian, *n* = 489; East Asian, *n* = 504; Admixed American, *n* = 347; European, *n* = 503; and African, *n* = 661. The 21 ACE2 genetic polymorphisms and NES are listed below: rs142239085 (0.4), rs75979613 (−0.44), rs144734382 (−0.3), rs12010448 (0.43), rs12006793 (−0.1), rs432654 (0.22), rs62578874 (0.32), rs112171234 (−0.85), rs5935990 (0.4), rs6629110 (0.25), rs147214574 (0.2), rs192766593 (−0.24), rs5936003 (0.2), rs4830543 (0.38), rs1996225 (0.27), rs112504565 (0.41), rs12558179 (0.15), rs5980185 (−0.16), rs73202884 (−0.14), rs11798628 (0.19), rs143695310 (0.17).

### 2.3. Polymorphism Analysis of 3′UTR, 5′UTR, and Promoter Regions in ACE2

Each ACE2 genetic polymorphism in the 3′UTR, 5′UTR and promoter regions (promotor region is defined as ~2 kb upstream of the start codon) was analyzed. Allele frequencies of more than 0.01 were selected as rare allele frequencies that are difficult to screen for future case-control genetic studies. Allele frequencies were obtained from 1000 Genomes Project, Exome Aggregation Consortium, and Genome Aggregation Database on dbSNP on the National Center for Biotechnology Information (NCBI) website (https://www.ncbi.nlm.nih.gov/snp/).

## 3. Results

### 3.1. Dynamic Tracking of SARS-CoV-2 S Glycoprotein D614G Mutation

**Figure 1:**
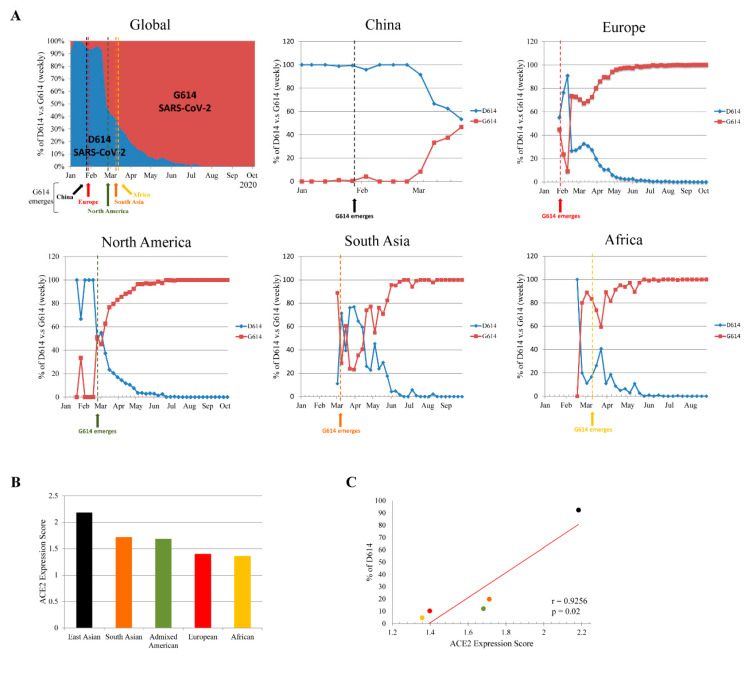
Transition of severe acute respiratory syndrome coronavirus 2 (SARS-CoV-2) spike (S) glycoprotein D614 to G614 variant in different geographic regions. (**A**) Weekly ratio of D614 and G614 variants globally, and in China, Europe, North America, South Asia, and Africa. Data analyzed from COVID-19 Viral Genome Analysis Pipeline (https://cov.lanl.gov/). (**B**) Angiotensin 1-converting enzyme 2 (ACE2) expression level in different geographical regions. ACE2 expression score analyzed by 21 ACE2 genetic polymorphisms that significantly affect its expression level according to the current study [18]. (**C**) Pearson’s correlation coefficient (r) and *p* value between ACE2 expression score and prevalence of D614 variants in different geographical regions.

To understand the global dynamic of SARS-CoV-2 D614G mutation frequencies, we analyzed 121,895 SARS-CoV-2 sequences from 1 January to 12 October 2020 from the Los Alamos National Laboratory website (COVID-19 Viral Genome Analysis Pipeline [12], https://cov.lanl.gov/; Figure 1A). In China, the D614 variant remained predominant from the end of December 2019 to March 2020. Though the G614 variant emerged in China on 28 January 2020, it did not reach an equal circulating ratio until 29 March to 4 April 2020. The emergence of the G614 variant in Europe and North America occurred on 29 January and 28 February 2020, respectively. The D614 and G614 variants reached equal ratio in Europe and North America around 17–23 February and 24 February to 1 March 2020, respectively. Immediately after reaching equal ratio, the G614 variant became the predominate circulating variant in both Europe and North America. The emergence of the G614 variant in South Asia and Africa occurred later, on 10 and 13 March, respectively. The D614 and G614 variants reached equal ratio in South Asia around 16–22 March. However, by the time the G614 variant first emerged in South Asia and Africa, the G614 variant was already the global circulating variant. Therefore, the evolutionary adaptation of the D614G mutation may have occurred in Europe or North America. The G614 variant emerged earlier in China than in other regions, but did not become the predominant variant, indicating that the D614 variant was adapted to this population. Notably, the D614 and G614 variants reached equal ratio in China after it became the predominant variant in the severe outbreak in Europe and North America, suggesting that the G614 variant may have been transmitted back to China after 29 March.

### 3.2. Genetic Variability in ACE2 Expression

These results have raised concern as to why the SARS-CoV-2 G614 variant became globally predominant without treatment and vaccine selective pressure. ACE2 plays an important role in SARS-CoV-2 attachment and is involved in the first step of viral infection. Variations in ACE2 expression could determine the efficiency of viral infection and replication, and thereby susceptibility to SARS-CoV-2. Recent reports demonstrated that ACE2 expression is significantly higher in Asian populations than that in to European, Admixed American, and African populations. Of these, African populations showed the lowest ACE2 expression level [18,21]. Figure 1B shows reanalysis of the ACE2 expression level (analysis from 21 ACE2 genetic polymorphisms that significantly affect protein expression level according to a previous study [18]) in different populations from the 1000 Genomes Project Phase 3 release database [20]. This again suggested that ACE2 expression is higher in East Asian populations than that in others. Moreover, correlation-coefficient analysis showed that there is a significant positive relationship between ACE2 expression and the prevalence of the D614 variant in different geographic regions (Figure 1C). This indicated that differences in ACE2 expression across geographic regions is a driving force for the positive selection of the SARS-CoV-2 S glycoprotein D614G mutation.

**Figure 2:**
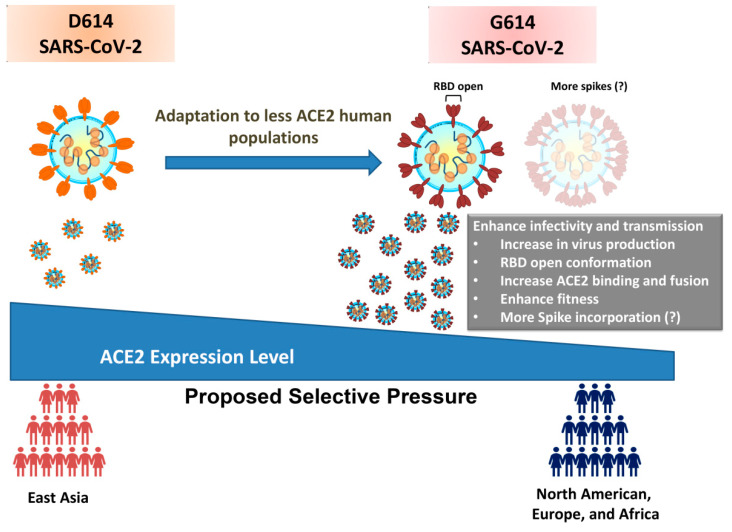
Genetic variability in ACE2 expression as potential driving force for presence of D614G mutation in SARS-CoV-2 S glycoprotein.

Another report found 2 ACE2 intron variants and 10 other protein intron variants located within or near the ACE2 gene (3 from *CLTRN*, 5 from *CA5B*, and 2 from an unknown gene) to be associated with higher ACE2 expression levels by analysis of expression quantitative trait loci (eQTLs) [22]. Of these 12 intron variants, 9 showed significantly higher allele frequencies in Asian populations when compared to those of others (African, European, and American; Table 1). Considering the critical roles of 3′UTR, 5′UTR, and promoter in protein expression, we further analyzed the allele frequencies of variants in the aforementioned regions of ACE2 gene. All ACE2 3′UTR and 5′UTR variants were rare allele frequencies; however, 8 ACE2 promoter variants showed significantly higher allele frequencies in African populations than in Asian, European, and American populations. No variants existed in Asian populations (Table 1). It is still unclear whether these differences in the allele frequencies of the promoter region play any role on ACE2 expression. According to the current evidence, the ACE2 genetic variants with high allele frequencies are associated with a higher expression level of ACE2 in Asian populations. In addition, the SARS-CoV-2 variant containing the D614G mutation is predominantly transmitted outside of China. Taken together, we speculate that the lower ACE2 expression in European and North American populations is a result of this genetic variation, and provides the driving force for the positive selection of SARS-CoV-2 S glycoprotein D614G mutation. The SARS-CoV-2 S glycoprotein D614G mutation may be selected as a result of enhanced transmission ability in populations with lower ACE2 expression (Figure 2).

## 4. Discussion

In this study, we provided a possible explanation for the positive selection of the D614G mutation in SARS-CoV-2. Evidence showed that the S glycoprotein D614G mutation could enhance the infectivity of SARS-CoV-2 by incorporating more spike proteins to the viral envelope, which thereby increases the chance of viral attaching in populations with lower expression of ACE2 in host cells. Recent evidence also showed that the SARS-CoV-2 S glycoprotein G614 mutation enhances the viral load in the nasal washes and trachea of hamsters, and may increase transmission ability [11]. The ACE2 binding site in S glycoprotein is partially shielded in closed conformation, hence affecting the binding [23,24]. The open conformation of the S glycoprotein could be required for binding with ACE2 and fusion. A molecular virological study investigated the details of conformational changes between D614 and G614 variants, and showed that the D614G mutation shifts the S glycoprotein conformation to be more open, which could contribute to the increased efficiency of ACE2 binding and fusion [10]. The SARS-CoV-2 D614G mutation notably did not increase the binding affinity with ACE2 [9,11].

The D614G mutation in S glycoprotein is a potential example of how positive selection drives the adaptive evolution of SARS-CoV-2. Rapid cumulative diversity could enhance the ability of SARS-CoV-2 to pass the barrier of genetic variation in different populations. There are some limitations in this study: primarily, it is not clear how the ACE2 expression score correlates with fold changes in ACE2 expression in different populations. It is also unclear which threshold of ACE2 expression score is required to provide enough driving force for SARS-CoV-2 selection. It seems as though the difference in ACE2 expression score between East Asian and North American populations is enough to provide selective pressure for the SARS-CoV-2 evolution. However, the NES is computed as the effect of the alternative allele relative to the reference allele, so the magnitude has no direct biological interpretation. Nonetheless, on the basis of current evidence, we demonstrated that there is strong correlation between ACE2 expression, and the dynamic of D614 and G614 variants across geographic regions. Moreover, we provide a future direction for investigating the relationship between biological differences and SARS-CoV-2 evolutionary adaption. Further studies using reverse genetics are required to confirm the relevance of our findings, connecting the D614G mutation with different ACE2 expression levels. Other factors, such as the host immune selective pressure, cannot be ruled out as important driving forces in SARS-CoV-2 evolution. SARS-CoV-2 may be seasonal, like other coronaviruses; therefore, continued monitoring of SARS-CoV-2 genetic diversity and understanding its effects on individual susceptibility are important to adopt a precision-medicine strategy for COVID-19 patients or high-risk individuals. Moreover, understanding the role of genetic diversity could improve clinical outcomes and decrease SARS-CoV-2 transmission. A large-scale genetic case control study with SARS-CoV-2 genome sequences, individual genetic variation, and clinical characterizations across geographic regions could provide a comprehensive understanding of the COVID-19 pandemic. 

It is still controversial as to whether patients receiving ACE inhibitors or angiotensin-receptor blockers could show enhanced susceptibility to SARS-CoV-2 infection or COVID-19 severity through increased ACE2 expression or attenuated inflammation and fibrosis, respectively. Since there are different levels of ACE2 expression among populations, it is important to examine whether different basal levels of ACE2 expression could affect susceptibility to SARS-CoV-2 infection or COVID-19 severity of patients receiving these drugs. Since SARS-CoV-2 is undergoing adaptive evolution, future studies for vaccine development and evaluation should carefully consider different levels of ACE2 expression among human populations, as there already potentially exists a certain number of SARS-CoV-2 viral quasispecies with higher S glycoprotein positive selective mutations in human populations that express lower levels of ACE2. Continuing to monitor evolutionary changes of SARS-CoV-2 in different populations is important to provide guidance on controlling and measuring public health in response to viral protein functional changes in the infectivity and severity of COVID-19.

## Figures and Tables

**Table 1:** Summary of population allele frequencies of host protein genetic variants related to ACE2 protein expression.

Rs ID	Ref. Allele	Variant	Function Class (a.a. Change)	Global	African	Asian	European	American	Other	Potential Functional Effect	Ref.
rs112171234	T	G	Intron	0.058	0.2047	0	0.00076	0.0245	0.025	Higher expression	[22]
rs4646127	A	G	Intron	0.75103	0.8009	0.9965	0.63994	0.767	0.701	Higher expression	[22]
rs4830974	G	A	5′ distal regions (*CLTRN* intron) ^†^	0.65138	0.73245	0.9396	0.50745	0.713	0.564	Higher expression	[22]
rs6632704	C	A	5′ distal regions (*CLTRN* intron) ^†^	0.58884	0.5088	0.9415	0.50677	0.699	0.535	Higher expression	[22]
rs2158082	G	A	5′ distal regions (*CLTRN* intron) ^†^	0.72834	0.95155	0.9955	0.50595	0.746	0.586	Higher expression	[22]
rs1996225	C	T	5′ distal regions (*CA5B* intron) ^†^	0.51673	0.57974	0.74372	0.41528	0.60129	0.4926	Higher expression	[22]
rs5936029	C	T	5′ distal regions (*CA5B* intron) ^†^	0.70741	0.88315	0.9875	0.50442	0.726	0.587	Higher expression	[22]
rs4830983	A	C	5′ distal regions (*CA5B* intron) ^†^	0.72881	0.95195	0.9885	0.51156	0.746	0.585	Higher expression	[22]
rs5936011	C	T	5′ distal regions (*CA5B* intron) ^†^	0.70679	0.88175	0.9875	0.50893	0.734	0.574	Higher expression	[22]
rs4060	C	A	5′ distal regions (*CA5B* intron) ^†^	0.63257	0.6688	0.9395	0.50713	0.708	0.548	Higher expression	[22]
rs6629110	T	C	5′ distal regions (Intergene region) ^†^	0.57021	0.43585	0.9575	0.50413	0.6875	0.526	Higher expression	[22]
rs143695310	T	A	5′ distal regions (Intergene region) ^†^	0.0174	0.0028	0	0.03528	0.022	0.034	Higher expression	[22]
rs4646112	C	T	Promoter	0.01921	0.06575	0	0.00012	0.013	0.009	Not characterized	-
rs4646113	G	A	Promoter	0.02078	0.07485	0	0.00058	0.0055	0.013	Not characterized	-
rs4646114	C	T	Promoter	0.01959	0.0667	0	0.00012	0.013	0.009	Not characterized	-
rs111572878	T	C	Promoter	0.05531	0.20065	0	0.00073	0.0155	0.023	Not characterized	-
rs113208650	C	T	Promoter	0.02839	0.1031	0	0.00065	0.0055	0.014	Not characterized	-
rs185721534	A	G	Promoter	0.02512	0.0905	0	0.00012	0.009	0.009	Not characterized	-
rs113208650	C	T	Promoter	0.02839	0.1031	0	0.00065	0.0055	0.014	Not characterized	-
rs113009615	A	delA	Promoter	0.05618	0.20125	0	0.00077	0.0165	0.023	Not characterized	-

^†^ Variant location is defined as relative to ACE2 gene loci. Promoter region of ACE2 is defined as ~2 kb upstream of start codon and selected allele frequencies more than 0.01. Allele frequencies obtained from 1000 Genomes Project, Exome Aggregation Consortium, and Genome Aggregation Database on dbSNP on the National Center for Biotechnology Information (NCBI) website (https://www.ncbi.nlm.nih.gov/snp/).
